# Complete genome sequence of two *Hominenteromicrobium mulieris* strains CIP 112194 and CIP 112527, isolated from a human fecal sample

**DOI:** 10.1128/mra.01229-24

**Published:** 2025-04-07

**Authors:** Gérald Touak, Damien Rei, Dominique Clermont, Christiane Bouchier

**Affiliations:** 1 CIP, CRBIP, DT, Microbiology Department, Institut Pasteur, Paris, France; 2 NBX Biosciences, SAS, Centre ADVISOR, Montpellier, France; University of Maryland School of Medicine, Baltimore, Maryland, USA

**Keywords:** gut microbiome, anaerobes, genomes

## Abstract

The human gut microbiota is dominated by obligately anaerobic bacteria. A method based on species-targeted sorting using flow cytometry under anaerobic conditions has been performed to analyze the gut microbiome. This report describes the complete genome sequences of two *Hominenteromicrobium mulieris* strains CIP 112194 and CIP 112527 that were isolated from the feces of a healthy adult from France. This volunteer is a young adult having a vegetarian diet.

## ANNOUNCEMENT

To study microbial communities, Afrizal et al. (1) developed an application of single-cell dispensing, which allows label-free sorting of microscopic particles. Based on this approach, 82 human gut bacterial strains have been characterized, including novel species such as *Hominenteromicrobium mulieris* (1). We report here the complete genome sequences of two strains of *H. mulieris* CIP 112194 and CIP 112527 isolated from human feces belonging to the ancillary cohort “COSIMMGEN J” initiated for the study and approved by the COMITE DE PROTECTION DES PERSONNES Ile de France 1 - DOSSIER: 2018-fév. -14819. These strains were isolated from a healthy volunteer’s fecal sample, targeted by two polyclonal antibodies against two *Faecalibacterium prausnitzii* phylogroups and sorted using flow cytometry under anaerobic conditions (2). They were grown in pre-reduced tryptone, peptone, glucose yeast extract medium (supplemented with 0.1% l-cysteine–HCl and vitamins) (3) 48 h at 37°C and under strict anaerobic conditions (5% H_2_/5% CO_2_/90% N_2_). Further analysis using 16S rRNA gene sequencing assigned the isolates to *Hominenteromicrobium* genus, which belongs to the *Oscillospiraceae* family along with *F. prausnitzii*.

Genomic DNA used for Illumina and Nanopore sequencing was extracted from an 8 mL overnight culture of the strains CIP 112194 and CIP 112527. Genomic-tip 100G kit (Qiagen) and Nanobind CBB kit (Pacific Biosciences) were used for CIP 112194 and CIP 112527, respectively. Illumina sequencing was performed by the Mutualized Platform for Microbiology (Institut Pasteur, Paris, France) following their standard workflow for library preparation (Nextera tagmentation kit, Illumina) and an Illumina NextSeq 550 device using a 2 × 150 bp protocol. Paired-end reads were trimmed using fqCleanerER v23.12 workflow with a Phred quality score of 25 and read length >= 100 bases (4). The same gDNA aliquots were also used for long-read sequencing using the ligation sequencing gDNA kit (SQK-NBD114.24, Oxford Nanopore Technologies). gDNAs were not sheared or size selected to perform libraries. Libraries were sequenced on a MinION Mk1C device for 48 h (Oxford Nanopore Technologies, UK) with R10.4 flow cell (FLO-MIN114, Oxford Nanopore Technologies). The Dorado v0.3.0 tool was used to perform base calling. A command-line tool Hybracter hybrid v0.7.3 has been used for *de novo* assembly method (5). Long reads are filtered and subsampled using Filtlong v0.2.1 with parameter “--min_quality 10 min_length 10000” for CIP 112527 and “--min_quality 10 min_length 5000” for CIP 112194. This retained 11,389 reads (175,875,634 bp with an N50 of 15,403 bp) and 41,843 reads (345,761,802 bp with an N50 of 8,351 bp) for CIP 112527 and CIP 112194, respectively. Genome assemblies were generated using Flye v2.9 yielding a single contig, marked as circular by Flye, for each strain. The contigs were finally polished using Illumina-trimmed paired-end reads with pypolca v0.3.1 for CIP 112194 and polypolish v0.6.0 for CIP 112527 (6). The contigs were reoriented to begin by the dnaA chromosomal replication initiator gene using dnaapler v0.7.0 (7).

Both genome assemblies consist of a single circular chromosome with a length of ~3,1 Mbp and a G+C content of 52.4% (Table 1). More information is shown in Table 1. A pairwise genome comparison between the draft genome of *H. mulieris* type strain CLA-AAH250 (GCF_020687165) and genome sequences of CIP 112194 and CIP 112527 reported an average nucleotide identity based on MUMmer of 98.88% (with 85.37% aligned nucleotides) and 98.98% (with 84.03% aligned nucleotides), respectively, (8) indicating that the two strains belong to *Hominenteromicrobium mulieris* species.

The annotation of the genomes was performed using the NCBI Prokaryotic Genome Annotation Pipeline (version 6.7, March 2024).

**TABLE 1:** Summary of DNA sequence assembly data

Feature	Data per strain	
	CIP 112194	CIP 112527
Sequencing statistics		
Number of filtered ONT reads	41,843	11,389
Filtered ONT read N50	8,351	15,403
Genome statistics		
Genome size (bp)	3,146,805	3,184,959
GC content (%)	52.4	52.41
No. of tRNAs	57	57
No. of rRNAs	6	6
Average coverage (×)^*a*^	108	54
Average coverage (×)^*b*^	284	461
No. of CDSs^*c*^	3,030	3,088
Accession no.	CP173189	CP173188
Sequence read archive accession		
Filtered ONT reads	SRR31246135	SRR31246133
Illumina paired-end reads	SRR31246134	SRR31246132

^*a*^ Coverage after subsampling of length and quality-filtered ONT reads using Filtlong (see text for details).

^*b*^ Coverage after polishing with Illumina paired-end reads (see text for details).

^*c*^ CDSs, coding sequences.

## Data Availability

The collection of volunteer’s samples has been reviewed by the COMITE DE PROTECTION DES PERSONNES Ile de France 1 - N°IRB / IORG #: IORG0009918 - N° ID-RCB: 0-A01353-36. The genome sequences were deposited at GenBank under the accession numbers CP173188 and CP173189. The Illumina paired-end reads can be found at SRA accession numbers SRR31246134 and SRR31246132. The ONT-filtered reads from the MinION MK1C run can be found at SRA accession numbers SRR31246135 and SRR31246133.
